# Associations between renal impairment and anemia in older, rural Japanese men: the Nagasaki Island study

**DOI:** 10.1186/1880-6805-33-7

**Published:** 2014-04-17

**Authors:** Yuji Shimizu, Shimpei Sato, Jun Koyamatsu, Hirotomo Yamanashi, Mako Nagayoshi, Koichiro Kadota, Mami Tamai, Kazuhiko Arima, Hironori Yamasaki, Yosuke Kusano, Noboru Takamura, Takahiro Maeda

**Affiliations:** 1 Department of Community Medicine, Nagasaki University Graduate School of Biomedical Science, Nagasaki, Japan; 2 Department of Island and Community Medicine, Nagasaki University Graduate School of Biomedical Science, Nagasaki, Japan; 3 Unit of Translational Medicine, Department of Immunology and Rheumatology, Nagasaki University Graduate School of Biomedical Science, Nagasaki, Japan; 4 Department of Public Health, Nagasaki University Graduate School of Biomedical Sciences, Nagasaki, Japan; 5 Center for Health and Community Medicine, Nagasaki University, Nagasaki, Japan; 6 Department of Internal Medicine, Nagasaki National Hospital, Nagasaki, Japan; 7 Department of Global Health, Medicine and Welfare, Nagasaki University Graduate School of Biomedical Sciences, Atomic Bomb Disease Institute, Nagasaki, Japan

**Keywords:** GFR, Anemia, Renal impairments, CKD, Men

## Abstract

**Background:**

Renal impairment is known to be associated with atherosclerosis, which in turn is reported to be positively associated with hemoglobin levels. In addition, renal impairment is known to be associated with a form of anemia known as renal anemia.

**Methods:**

To clarify the associations between renal impairment and anemia, we conducted a cross-sectional study of 1,105 60 to 89-year-old men, who were not taking medication for anemia and were undergoing general health check-ups.

**Results:**

Compared with non-chronic kidney disease, chronic kidney disease (CKD) with a glomerular filtration rate (GFR) <60 mL/min/1.73 m^2^ was found to constitute a significant risk of anemia. However, we noted that this risk was lower for mild renal impairment (60 mL/min/1.73 m^2^ ≤ GFR <90 mL/min/1.73 m^2^). Compared with the non-CKD reference group, the classical cardiovascular risk factors adjusted odds ratio (OR) for anemia was 1.81 (1.23 to 2.68) and compared with the normal renal function (GFR ≥90 mL/min/1.73 m^2^) reference group, the ORs for mild renal impairment and CKD were 0.26 (0.15 to 0.47) and 0.60 (0.33 to 1.09).

**Conclusions:**

Independent from classical cardiovascular risk factors, CKD, which was identified during general health check-ups, appeared to constitute a significant risk of anemia for older Japanese men. For mild renal impairment, however, this association was a reduced risk of anemia and thus possibly a higher risk of atherosclerosis.

## Introduction

A decline in the glomerular filtration rate (GFR) was identified as a marker of chronic kidney disease (CKD) that is known to be associated with a form of anemia known as renal anemia.

In a previous study of ours, positive associations were detected between hemoglobin levels and increased arterial stiffness assessed with the cardio-ankle index (CAVI) [1]. Since CAVI was identified as an independent factor associated with GFR in the general population [2], renal impairment might also be positively associated with hemoglobin levels.

To investigate this potential association, we conducted a cross-sectional study of older Japanese men who participated in a general health check-up between 2005 and 2012.

## Methods

### Subjects

The survey population covered 1,175 men aged 60 to 89 years, who were all residents of the western Japan rural community of the Goto Islands and participated in this study between 2005 and 2012. We used data that were obtained the first time the subjects participated in this study. A total of 66 individuals with missing data and 4 individuals who were taking medication for anemia were excluded, leaving 1,105 men for enrollment in this study. The mean age of the study population was 70.2 years (±6.7 SD; range 60 to 89). Written consent forms were available in Japanese to ensure comprehensive understanding of the study objectives, and informed consent was signed by the participants. This study was approved by the Ethics Committee for Use of Humans of Nagasaki University (project registration number 0501120073).

### Data collection and laboratory measurements

Systolic blood pressures were recorded at rest. Body weight and height were measured with an automatic body composition analyzer (BF-220; Tanita, Tokyo, Japan) at the same time blood was drawn. Trained interviewers obtained information on smoking status, drinking status, medical history, use of antihypertensive agents, and use of medication for diabetes mellitus. Fasting blood samples were obtained in an EDTA-2 K tube and were used for measuring hemoglobin with the sodium lauryl sulfate (SLS)-hemoglobin method. Serum concentrations of HDL cholesterol, triglycerides (TG), aspartate aminotransferase (AST), γ-glutamyltraspeptidase (γGTP), creatinine, and hemoglobin A1c (HbA_1c_), were measured with standard laboratory procedures. The glomerular filtration rate (GFR) was estimated by using an established method with three variations recently proposed by a working group of the Japanese Chronic Kidney Disease Initiative [3].

### Definition of disease

Anemia for men was defined as 13 g/dL > hemoglobin in accordance with the recommendation by the World Health Organization (WHO) Study Group [4]. Diabetes was defined as HbA_1c_ ≥6.5% (NGSP: National Glycohemoglobin Standardization Program) and/or initiation of glucose-lowering medication or insulin therapy [5]. On the basis of the Kidney Disease Outcomes Quality Initiative Clinical Practice Guidelines [6], we defined normal renal function as GFR ≥90 mL/min/1.73 m^2^, reduced renal function as GFR <90 mL/min/1.73 m^2^, mild renal impairment as GFR 60 to 89 mL/min/1.73 m^2^, and CKD as GFR <60 mL/min/1.73 m^2^.

### Statistical analysis

Differences in age-adjusted mean values or prevalence of potential confounding factors in relation to hemoglobin levels were calculated using ANOVA or logistic regression models. Odds ratios (ORs) and 95% confidence intervals (CIs) for anemia associated with renal impairment (both mild renal impairment and CKD) were calculated with the aid of logistic regression models. First, we adjusted only for age. Second, we included other potential confounding factors, that is, smoking status (never a smoker, former smoker, current smoker), alcohol consumption (non-drinker and current light to moderate drinker (1 to 6 times/week), current heavy drinker (every day)), systolic blood pressure (mmHg), antihypertensive medication use (no, yes), body mass index (kg/m^2^), diabetes (no, yes), history of cardiovascular disease (no, yes), HDL cholesterol (mg/dL), TG (mg/dL), AST (IU/L), and γGTP (IU/L). All statistical analyses were performed with the SAS system for Windows (version 9.3; SAS Inc., Cary, NC, USA). All *P* values for statistical tests were two-tailed, and values of <0.05 were regarded as statistically significant.

## Results

Of the 1,105 men taking part in the general health check-up program, 645 were diagnosed with mild renal impairment and 351 with CKD, while 162 were diagnosed with anemia.

Table 1 lists age-adjusted characteristics for this population in relation to GFR levels.

**Table 1:** Age-adjusted mean values and percentage in relation to renal function

	**Non-chronic kidney disease**^**d**^		**Chronic kidney disease**^**c**^	***P***
	**Normal renal function**^**a**^	**Mild renal impairment**^**b**^		
Number at risk	109	645	351	
Age, years	67.7 ± 6.1	69.6 ± 6.5	72.2 ± 6.7	
Systolic blood pressure, mmHg	143	143	145	0.344
Antihypertensive medication use, %	31.5	34.3	40.6	0.083
Diabetes, %	14.0	12.0	10.7	0.634
History of cardiovascular disease, %	8.8	10.2	17.4	0.003
Body mass index, kg/m^2^	22.8	23.5	23.9	0.003
Current drinkers, %	56	46.8	51.1	0.126
Current smokers, %	29.1	21.6	15.2	0.004
Serum HDL cholesterol, mg/dL	57	55	52	0.004
Serum triglycerides (TG), mg/dL	116	112	135	<0.001
Serum aspartate aminotransferase (AST), IU/L	27	25	24	0.021
Serum γ-glutamyltraspeptidase (γGTP), IU/L	44	37	46	0.046
Serum creatinine, mg/dL	0.62	0.82	1.18	<0.001
Glomerular filtration rate (GFR), mL/min/1.73 m^2^	99.6	72.4	49.8	<0.001

^a^Normal renal function, ^b^mild renal impairment, and ^c^chronic kidney disease were defined as respectively, GFR ≥90 mL/min/1.73 m^2^, 90 mL/min/1.73 m^2^ > GFR ≥60 mL/min/1.73 m^2^, and GFR <60 mL/min/1.73 m^2^. ^d^Non-chronic kidney disease includes normal renal function and mild renal impairment.

We found that compared with non-CKD, CKD constituted a significant risk factor for anemia (Table 2). However, when we divided the non-CKD group into a normal renal function group (GFR ≥90 mL/min/1.73 m^2^) and a mild renal impairment group (60 < GFR ≤90 mL/min/1.73 m^2^), and used the normal renal function group for reference, mild renal impairment was found to be associated with a significantly lower risk of anemia. CKD was not associated with a higher risk of anemia.

**Table 2:** Odds ratios (OR) and 95% confidence intervals (CI) for anemia in relation to glomerular filtration rate (GFR) levels

	**Non-chronic kidney disease**^**d**^			**Chronic kidney disease**^**c**^	***P***
	**Normal renal function**^**a**^		**Mild renal impairment**^**b**^		
Number at risk	109		645	351	
Number of cases (percentage)	26 (23.9)		63 (9.8)	73 (20.8)	
Age-adjusted OR		1.00		1.54 (1.08 to 2.20)	0.016
	1.00		0.26 (0.16 to 0.45)	052 (0.30 to 0.89)	0.967
Multivariable OR		1.00		1.81 (1.23 to 2.68)	0.003
	1.00		0.26 (0.15 to 0.47)	0.60 (0.33 to 1.09)	0.644

^a^Normal renal function, ^b^mild renal impairment, and ^c^chronic kidney disease (CKD) were defined as respectively, GFR ≥90 mL/min/1.73 m^2^, 90 mL/min/1.73 m^2^ > GFR ≥60 mL/min/1.73 m^2^, and GFR <60 mL/min/1.73 m^2^. ^d^Non-chronic kidney disease includes normal renal function and mild renal impairment. Anemia: hemoglobin in level <13.0 g/dL. Multivariable OR: adjusted further for age, systolic blood pressure, antihypertensive medication use, body mass index, smoking, alcohol intake, diabetes, history of cardiovascular disease, serum HDL cholesterol, serum triglycerides (TG), serum aspartate aminotransferase (AST), and serum γ-glutamyltranspeptidase (γGTP).

Participants with CKD and anemia showed significantly lower GFR whereas their non-CKD counterparts showed significantly higher GFR. The age-adjusted values for GFR were 50.6 mL/min/1.73 m^2^ for participants with non-anemic CKD and 44.8 mL/min/1.73 m^2^ for those with anemic CKD (*P* <0.001). For non-CKD participants, the corresponding values were 75.5 mL/min/1.73 m^2^ and 84.3 mL/min/1.73 m^2^ (*P* <0.001).

As part of our study, we conducted a further investigation of 1,059 men for whom CAVI data were available. We found that both GFR and hemoglobin were significantly associated with atherosclerosis (CAVI ≥9.0). The age-adjusted odds ratios for risk of atherosclerosis of a one standard deviation (SD) increment in GFR (17.3 mL/min/1.73 m^2^) and in hemoglobin (1.3 g/dL) were 0.82 (0.71 to 0.95, *P* = 0.007) and 1.31 (1.14 to 1.50, < 0.001), respectively.

## Discussion

The major finding of the study presented here was that CKD, which was diagnosed during general health check-ups, seems to represent a significant risk of anemia for older, rural Japanese men. Since this association was the result of the reduced risk of anemia for mild renal impairment, which might be associated with atherosclerosis, this finding may constitute an important tool for clinical practice dealing with renal impairment.

A previous study of ours reported that hemoglobin levels were positively associated with hypertension [7] and with increased arterial stiffness as evaluated by CAVI [1] for non- anemic men.

**Figure 1:**
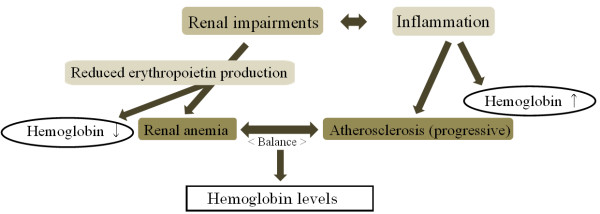
Association between renal impairment and anemia.

Findings of our analyses showed that CKD constitutes a significant risk of anemia when compared with non-CKD, even though we also found that, when compared with normal renal function, CKD does not constitute a greater risk of anemia. Moreover, mild renal impairment proved to be associated with a significantly lower risk of anemia. These findings suggest that the positive association between CKD and anemia in comparison with non-CKD is not caused mainly by renal anemia but by the influence of the lower risk of anemia associated with mild renal impairment. Since the target population for our study consisted of healthy older Japanese men, the number of participants with CKD accompanied by anemia was relatively small: 73 with anemic CKD as compared with 278 with non-anemic CKD. However, a previous Japanese study of older men (aged ≥70 years) showed that the odds ratio for risk of anemia was significantly higher for a GFR of less than 90 mL/min/1.73 m^2^[8]. To clarify these discrepancies, we conducted a further analysis, which revealed that both GFR and hemoglobin were significantly associated with atherosclerosis (CAVI ≥9.0). The findings of this additional analysis also indicate that the association between renal condition and hemoglobin level may depend on the balance between atherosclerosis and renal anemia (Figure 1).

This study has certain potential limitations, which warrant consideration. One of these is that we could not establish any causal relationships because this study was cross-sectional. Since we consider the positive association between hemoglobin and renal impairment to be caused by active atherosclerosis progression, the atherosclerosis should also be classified into stable and progressive atherosclerosis. This indicates the need for a longitudinal study with a larger number of participants.

## Conclusions

In conclusion, CKD appeared to constitute a significant risk of anemia for older, rural Japanese men as determined during general health check-ups. For mild renal impairment, however, this association was the result of a reduction in the risk of anemia and thus possibly a higher risk of atherosclerosis.

## Abbreviations

AST: Aspartate aminotransferase; CAVI: Cardio-ankle index; CI: Confidence intervals; CKD: Chronic kidney disease; GFR: Glomerular filtration rate; HbA1c: Hemoglobin A1c; NGSP: National Glycohemoglobin Standardization Program; ORs: Odds ratios; SLS: Sodium lauryl sulfate; TG: Triglycerides; WHO: World Health Organization; γGTP: γ-glutamyltraspeptidase.

## Competing interests

The authors have no financial or any other kinds of conflicts in connection with this paper.

## Authors’ contributions

YS designed the study and performed the statistical analyses, interpreted the data, and drafted and revised the manuscript. MN, KK, SS and JK assisted with the design of the study, were involved in data collection, and checked the manuscript. HY, NT, MN, KA, MT, and YK participated in the study concept and checked the manuscript. TM was the general coordinator and also designed the study. All authors read and approved the final manuscript.

## References

[B1] Shimizu Y Nakazato M Sekita T Kadota K Yamasaki H Takamura N Aoyagi K Maeada T Association between hemoglobin levels and arterial stiffness for general Japanese population in relation to body mass index status: The Nagasaki Islands Study Geriatr Gerontol Int 2013 doi:10.1111/ggi.12171 10.1111/ggi.12171 24215101

[B2] Kubozono T Miyata M Ueyama K Nagaki A Hamasaki S Kusano K Kubozono O Tei C Association between arterial stiffness and estimated glomerular filtration rate in the Japanese general population J Atheroscler Thromb 2009 16 840 845 20032588 10.5551/jat.1230

[B3] Imai E Equation for estimating GFR from creatinine in Japan Nihon Rinsho 2008 66 1725 1729 18788401

[B4] Beutler E Waalen J The definition of anemia: what is the lower limit of normal of the blood hemoglobin concentration? Blood 2006 107 1747 1750 10.1182/blood-2005-07-3046 16189263 PMC1895695

[B5] American Diabetes Association Diagnosis and classification of diabetes mellitus Diabetes Care 2013 36 S67 S74 23264425 10.2337/dc13-S067 PMC3537273

[B6] National Kidney Foundation K/DOQI clinical practice guidelines for chronic kidney disease: elevation, classification, and stratification Am J kidney Dis 2002 39 S1 S266 11904577

[B7] Shimizu Y Nakazato M Sekita T Kadota K Arima K Yamasaki H Takamura N Aoyagi K Maeda T Association between the hemoglobin levels and hypertension in relation to the BMI status in a rural Japanese population: The Nagasaki Islands Study Intern Med 2014 53 435 440 10.2169/internalmedicine.53.1353 24583431

[B8] Kohagura K Tomiyama N Kinjo K Takishita S Iseki K Prevalence of anemia according to stages of chronic kidney disease in a large screening cohort of Japanese Clin Exp Nephrol 2009 13 614 620 10.1007/s10157-009-0197-z 19526304

